# Unsuitability of MALDI-TOF MS to discriminate *Acinetobacter baumannii* clones under routine experimental conditions

**DOI:** 10.3389/fmicb.2015.00481

**Published:** 2015-05-19

**Authors:** Clara Sousa, João Botelho, Filipa Grosso, Liliana Silva, João Lopes, Luísa Peixe

**Affiliations:** ^1^Centro de Engenharia Biológica, Universidade do MinhoBraga, Portugal; ^2^UCIBIO/REQUIMTE, Laboratório de Microbiologia, Departamento de Ciências Biológicas, Faculdade de Farmácia, Universidade do PortoPorto, Portugal; ^3^Departamento de Farmácia Galénica e Tecnologia Farmacêutica, Faculdade de Farmácia, Universidade de LisboaLisboa, Portugal

**Keywords:** *Acinetobacter baumannii*, typing, MALDI-TOF MS, subspecies, chemometrics, sequence type

## Abstract

MALDI-TOF MS (matrix-assisted laser desorption/ionization time-of-flight mass spectrometry) is now in the forefront for routine bacterial species identification methodologies, being its value for clonality assessment controversial. In this work we evaluated the potential of MALDI-TOF MS for assisting infection control by depicting *Acinetobacter baumannii* clones. Mass spectra of 58 *A. baumannii* clinical isolates belonging to the worldwide spread lineages (ST98, ST103, ST208, and ST218) isolated in our country, were obtained and analyzed with several chemometric tools (pseudo gel views, *peakfind* function, and partial least squares discriminant analysis). The clonal lineages were obtained using the “Oxford” scheme, belonging ST98, ST208, and ST218 to the international clone II and ST103 to an epidemic clonal lineage (SG5). Additionally, mass spectra of a highly diverse international collection of 38 isolates belonging to 22 sequence types (STs) were obtained for further comparisons. Pseudo gel views and direct peak pattern analysis did not allow the discrimination of *A. baumannii* isolates belonging to ST98, ST103, ST208, or ST218. Moreover, a partial least square discriminant analysis of the mass spectra considering two spectral ranges (2–20 kDa and 4–10 kDa) revealed a poor degree of discrimination with only 64.6 and 65.8% of correct ST assignments, respectively. Also, mass spectra of the international isolates (*n* = 38, 22STs) revealed a very congruent peak pattern among them as well as among the four lineages included in this work. Despite the increasing interest of MALDI-TOF MS for bacterial typing at different taxonomical levels, we demonstrated, using routine experimental conditions, the unsuitability of this methodology for *A. baumannii* clonal discrimination.

## Introduction

During the last decade, the rate of nosocomial infections caused by multidrug-resistant *Acinetobacter baumannii* (MDRAB) has increased worldwide. In particular, the growing number of carbapenem-resistant *A. baumannii* isolates, mainly due to the production of carbapenem-hydrolyzing class D β-lactamases (CHDLs) jeopardizes the treatment of infections caused by this agent (Higgins et al., 2010). The quick and reliable clonality assessment is crucial to rapidly trace its dissemination, assist antibiotherapy, and implement measures to constrain its dissemination.

Pulsed-field gel electrophoresis (PFGE), amplified fragment length polymorphism (AFLP) analysis, multilocus sequence typing (MLST), Diversilab^TM^ typing, other PCR fingerprinting methods, and multiple-locus variable tandem repeat number analysis (MVLA) are among the current methods used for genotyping bacteria (Grosso et al., 2011b; Karah et al., 2012; Zander et al., 2012; Zarrilli et al., 2013). PCR and sequence-based methods which describe isolates numerically are easy to use in international networks. Specifically for *A. baumannii,* Sequence Groups (SGs) identification or Trilocus (Turton et al., 2007) sequence-based typing (3LST – selectively amplifies alleles of *omp*A, *csu*E, and *bla*_OXA-51_ genes), *bla*_OXA-51-like_ genotyping and two MLST schemes^1^ (“Oxford” and Institute Pasteur) are available. The 3LST, *bla*_OXA-51-like_ genotyping, and Diversilab^TM^ typing (which consists in a repetitive extragenic palindromic PCR – rep-PCR) allow the rapid identification of the main multidrug-resistant *A. baumannii* lineages, being already established the concordance between some of these methods (Zarrilli et al., 2013). The MLST schemes share three loci but reveal different discriminatory abilities, with our data pointing for a higher resolution, congruent to PFGE analysis and CHDL content, of the “Oxford” scheme (Grosso et al., 2011b). MLST analysis in several collections resulted in the recognition of major clonal complexes (CCs) and sequence types (STs) responsible for antimicrobial resistance dissemination, such as CC92 (ST92, ST208, ST218), CC109 (ST109), CC103 (ST103, ST133), and CC113 (ST113) according with the Oxford scheme (Zarrilli et al., 2013).

However, most of these methods are too expensive and/or time consuming. Spectroscopic techniques might constitute reliable alternatives for bacterial typing at different taxonomic levels, with variable degrees of success reported from their application to several microorganisms (Mencacci et al., 2013; Šedo et al., 2013; Vaz et al., 2013; Branquinho et al., 2014a,b; Novais et al., 2014; Sousa et al., 2014a). Recently, we developed and validated a mathematical model for typing *A. baumannii* clones, most of them included in this study, based on spectra obtained by a competitive spectroscopic technique, Fourier transform infrared spectroscopy with attenuated total reflectance (FTIR-ATR), that could be routinely used (Sousa et al., 2014b). Moreover, using matrix assisted laser desorption/ionization time-of-flight mass spectrometry (MALDI-TOF MS) and chemometrics we were able to circumvent difficulties associated with the discrimination by MALDI-TOF MS of *Acinetobacter* species within the *A. baumannii-calcoaceticus* complex (Böhme et al., 2010; Espinal et al., 2011; Sousa et al., 2014a). In which concerns the suitability of MALDI-TOF MS for bacterial typing at subspecies level, controversial data is available (Barbuddhe et al., 2008; Dieckmann and Malorny, 2011; Gekenidis et al., 2014; Lasch et al., 2014; Novais et al., 2014; Wolters et al., 2011).

In this work, we evaluated the ability of MALDI-TOF MS combined with several chemometric tools for assisting infection control by depicting *A. baumannii* clones.

## Materials and Methods

### Rational of the Study

To evaluate the potential of MALDI-TOF MS to discriminate *A. baumannii* clones, mass spectra of 58 Portuguese clinical isolates belonging to ST98, ST103, ST208, and ST218 according with the “Oxford” scheme were obtained and analyzed with several chemometric tools. Mass profiles analysis was performed by different approaches: (i) spectral overview on the basis of pseudo gel views; (ii) analysis of the mass peak pattern according the number of peaks found with the Matlab function *peakfind*; and (iii) partial least squares discriminant analysis (PLSDA). The last two approaches also allowed the estimative of correct predictions for each ST and the PLSDA model was performed considering the entire (2–20 kDa) and a selected (4–10 kDa) spectral range where the majority of the mass peaks (about 90%) were located. Additionally, mass spectra of 38 isolates from different countries and belonging to 22 distinct STs were obtained and the respective pseudo gel views compared among each other and with the ones of ST98, ST103, ST208, and ST218 isolates. We further explored a possible correlation between the mass profiles of the isolates tested and clustering/classification obtained with other methods for major lineages *A. baumannii* identification, as AFLP, SG typing, and the MLST scheme of Institute Pasteur.

### Bacterial Isolates

Fifty-eight *A. baumannii* clinical isolates were selected among a previously published collection of 318 CHDL-producing *Acinetobacter* spp. recovered from six geographically distant Portuguese hospitals (2001–2012). The selected isolates are representatives of the main lineages disseminated in Portugal before 2012: ST98 (*n* = 10); ST103 (*n* = 9); ST208 (*n* = 17; corresponding to a reassignment of ST92 isolates), and ST218 (*n* = 22; Grosso et al., 2011a,b; Sousa et al., 2014b). Additionally, 38 isolates belonging to 22 different STs were used for further comparisons. The main characteristics of the isolates used in this study, including the relationship between CHDL content, PFGE types, SGs, and the STs obtained according with Institute Pasteur and “Oxford” scheme^1^ among others were presented in **Table 1**.

**Table 1:** Epidemiological details of the *Acinetobater baumannii* isolates included in this study.

ST (CC)^b^	ST (CC)^c^	SG (AFLP)^a^	*n*^d^	PFGE^e^	CHDL^f^	Country	Years	Reference
98 (92)	2 (2)	SG1 (II)	10	A	OXA-40	Portugal	2001–2008	Grosso et al. (2011b), Sousa et al. (2014b)
208 (92)	2 (2)	SG1 (II)	17	A4	OXA-23	Portugal	2006–2010	Grosso et al. (2011b), Sousa et al. (2014b)
218 (92)	2 (2)	SG1 (II)	22	A5	OXA-23	Portugal	2010	Sousa et al. (2014b); This study
103 (103)	15 (15)	SG5	9	B, C	OXA-58	Portugal	2001–2004	Grosso et al. (2011b), Sousa et al. (2014b)
15		G1ompA/G2csuE	1	O	-	Germany	1991	Seifert et al. (2005), Sousa et al. (2014b)
16		SG2 (I)	1	L	-	Germany	1991	Seifert et al. (2005), Sousa et al. (2014b)
113 (113)	79 (79)	SG1 (II)	4	D	OXA-23	Brazil	2006–2007	Grosso et al. (2011a)
132	162	G1_OXA66_/G2_ompA_/_csuE_	2	F	OXA-23	Brazil	2007	Grosso et al. (2011a)
133 (103)	15 (15)	SG4	3	E	OXA-23	Brazil	2006–2007	Grosso et al. (2011a)
134	316	G2_ompA_/_OXA69_	1	K	OXA-23	Brazil	2007	Grosso et al. (2011a)
195		SG1 (II)	1	P	OXA-23	Germany	2010	Sousa et al. (2014b)
231		G2_OXA-69_/_ompA_	2	J, X	OXA-23	Germany, Italy	2004–2012	Sousa et al. (2014b)
236		SG1 (II)	1	T	-	Italy	2012	This study
348		SG1 (II)	1	R	OXA-72	Croatia	2001–2007	Goic-Barisic et al. (2011), Sousa et al. (2014b)
391		G1_ompA_	1	U	-	Germany	2012	Sousa et al. (2014b)
437 (92)	2 (2)	SG1 (II)	5	A4	OXA-58	Italy	2004–2008	D’Arezzo et al. (2009), Sousa et al. (2014b)
439		SG2 (I)	1	M	-	Germany	1991	Seifert et al. (2005), Sousa et al. (2014b)
441		SG2 (I)	4	H	-	Germany, Croatia	2001–2007	Sousa et al. (2014b)
513 (92)	2 (2)	SG1 (II)	2	A5	OXA-23	Italy	2007–2009	D’Arezzo et al. (2011), Sousa et al. (2014b)
514		G1_csuE_/G2_OXA-69_	1	N	-	Germany	1991	Sousa et al. (2014b)
515			1	I	-	Portugal	2010	Sousa et al. (2014b)
732		G1_ompA_	2	V, W	-	Germany	2012	Sousa et al. (2014b)
733		G1_ompA_	1	Y	OXA-23	Germany	2012	Sousa et al. (2014b)
740		SG2 (I)	1	Q	-	Croatia	2001–2007	Sousa et al. (2014b)
775		SG2 (I)	1	G	-	Croatia	2001–2007	Sousa et al. (2014b)
776		G1_OXA66_/_ompA_/G2_csuE_	1	S	-	Italy	2012	Sousa et al. (2014b)

^a^SG, sequence group typing (PCR-based sequence groups based on *ompA*, *csuE,* and *bla*_OXA-51_ genes); AFLP, amplified fragment length polymorphism correspondence to international clones I–III); ^b^Sequence Type (ST) and Clonal Complex (CC) determined according with the “Oxford” scheme; ^c^Sequence Type (ST) and Clonal Complex (CC) determined according with Institut Pasteur MLST scheme; ^d^*n* total of isolates; ^e^ApaI-Pulsed Field Gel Electrophoresis type; ^f^CHDL, acquired carbapenem-hydrolyzing class D β-lactamase.

### MALDI-TOF MS Experiments

Mass spectra were obtained from cell extracts prepared according the manufacturer instructions. Briefly, overnight cultures in Muller-Hinton agar were suspended in HPLC water and treated with ethanol (75%). After centrifugation and removal of the supernatant, cells were extracted with 25 μL of 70% formic acid followed by addition of 25 μL of acetonitrile and vortexing at 2000 rpm for 1 min. Samples were spotted onto MALDI ground steel target (AnchorChip) followed by drying and the addition of 1 μL of the chemical matrix (saturated solution of α-cyano-4-hydroxycinnamic acid in 50% of acetonitrile and 2.5% of trifluoroacetic acid). Spectra were randomly obtained from blind samples in the linear positive mode at a laser (nitrogen) frequency of 20 Hz in the range of 2–20 kDa with a Microflex III instrument (Bruker Daltonic, Bremen, Germany). Each recorded spectrum is the result of six series of 40 single laser shots in different locations. The experiments were performed in quadruplicate using four distinct spots of the MALDI target (instrumental replicates) at least in two different days (biological replicates) with two different bacterial cultures or extracts. External calibration of the mass spectra was performed using *Escherichia coli* DH5 alpha standard peaks (BTS).

### Data Analysis

Due to the large amount of data generated by MALDI experiments, mean spectrum for each isolate was generated from the instrumental and biological replicates and considered for further analysis. Zero-line and low S/N ratio mass spectra were not considered to the average. The pseudo gel views were generated by the dedicated Matlab-based software MicrobeMS^2^ that provides direct access to the spectra of Bruker’s proprietary file format. In these gel views the intensities are gray-scaled (log scale) being the mass/charge ratios (m/z) the abscissa and spectral indices the ordinate values. In these bar code spectra only the information of peak presence or absense is employed, while the peak intensity is neglected.

Mass spectra were also analyzed with the *peakfind* function of the PLS Toolbox for Matlab (arguments: 9- number of points in Savitzky–Golay filter, 6- tolerance on the estimated residuals; peaks heights are estimated to be >tolerance^∗^residuals) and 19- window width for determining local maxima to evaluate the intra and inter-ST variability among the four distinct STs. The method starts by estimating the peak mass-to-charge ratios of each ST. For this task, spectra of all isolates belonging to one ST were averaged, the result was submitted to the peak identification method and the peak locations were stored in a vector generating a “peak prototype” for each ST. This method was repeated for all STs. Then, the same peak identification method was run for each isolate individually. Peak positions of each “ST prototype” were compared with peak positions of each isolate. When a “ST prototype” peak location matched a peak location of an isolate, a value 1 was assigned for that peak; otherwise a value 0 was assigned. This procedure creates a vector of 0s and 1s for each pair “ST prototype”/isolate. Note that peak locations were considered to match if they were located within a mass-to-charge ratio difference lower than 7 m/z units [if for a certain peak location *n*, | m/z(prototype)_n_–m/z(sample)_n_| < 7, that peak is considered to match]. For each isolate, a percentage of matching peaks was estimated for each ST. Isolates were considered to belong to the ST yielding the highest percentage of peak matches.

For clustering purposes spectra were analyzed by PLSDA after the pre-processing mean-centring (Savitzky and Golay, 1964; Geladi and Kowalsky, 1986; Barker and Rayens, 2003). This pre-processing method allows removing the influence of different sample amounts and/or equipment variations in the peaks intensity. In PLSDA, to each known sample (*x_i_*) is assigned a vector of 0s with the value 1 at the position corresponding to its ST (*y_i_*). The structure of the PLSDA model is described by Eqs 1 and 2. Model loadings (*P* and *Q*) and corresponding scores (*T* and *U*) are obtained by sequentially extracting the components or latent variables (LVs) from matrices *X* (the spectra) and *Y* (the matrix codifying the STs).

(1) $$X={TP}^{t}+E$$

(2) $$Y={UQ}^{t}+F$$

The algorithm correlates the scores of each block (*T* and *U*), yielding an internal regression matrix. This internal regression can be transformed on a regression matrix (*B*). In this case, the regression matrix is composed by three vectors: one regression vector corresponding to each ST. *E* and *F* are the residual matrices and depend on the number of LV selected. Predictions for new samples are obtained by multiplying a new spectrum (*x_new_*) by the regression matrix (*B*).

(3) $$y_{new}=x_{new}B$$

The prediction (y_new_ = [y_new,1,_y_new,2,_...._,_y_new,n_]) is then converted in a class assignment. In PLSDA a probability value for each assignment is estimated for each sample. The model number of LV was optimized using the leave-one-sample-out cross-validation procedure in order to prevent overfitting. All chemometric models were performed in Matlab version 7.4 Release 2007a (MathWorks, Natick, MA, USA) and PLS Toolbox version 4.2.1 for Matlab (Eigenvector Research, Manson, WA, USA).

## Results

### Spectral Overview

**FIGURE 1:**
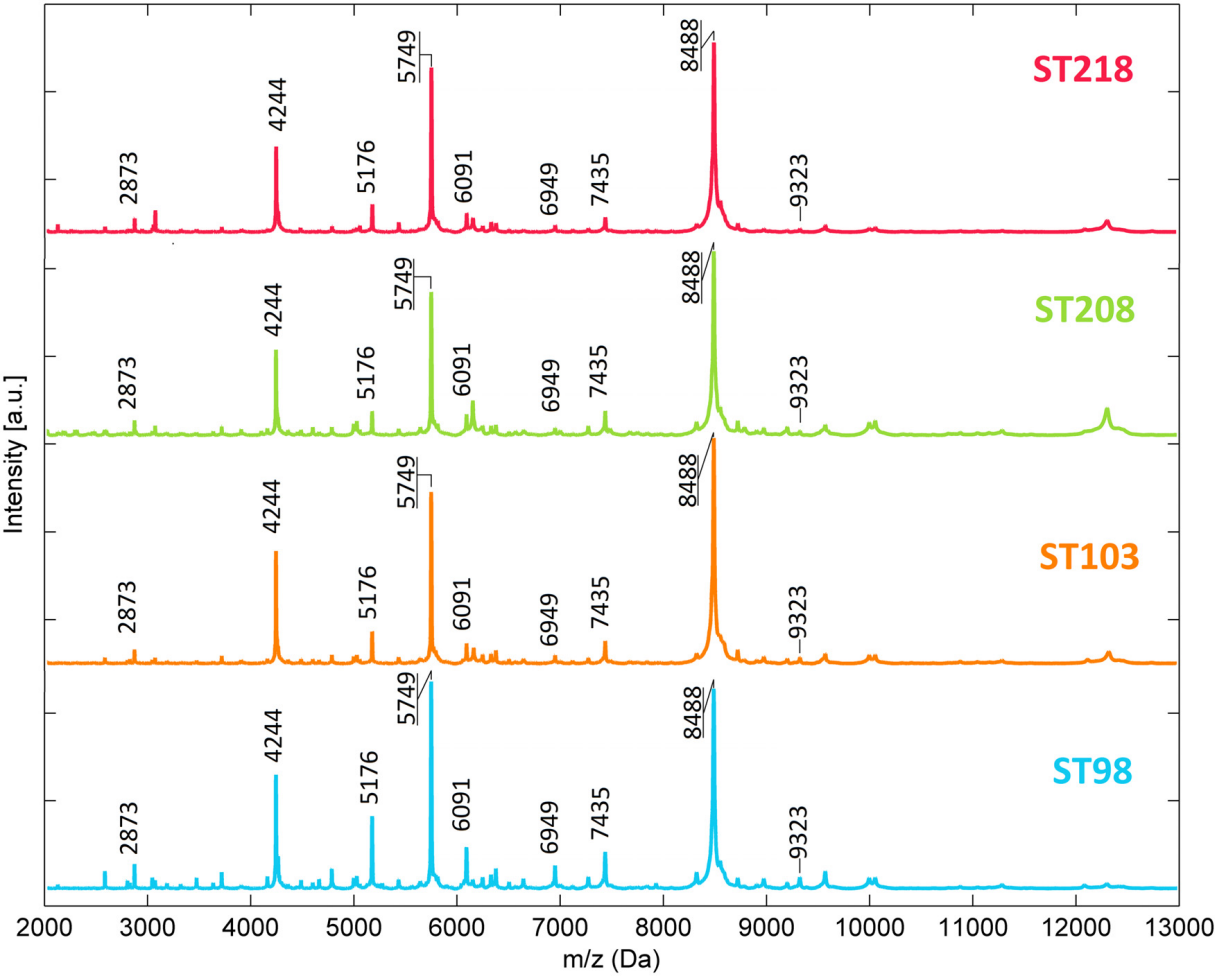
**Mass profiles of ST98, ST103, ST208, and ST218 isolates in the region of 2–13 kDa [obtained from the mean spectra of each sequence types (ST)]**.

**FIGURE 2:**
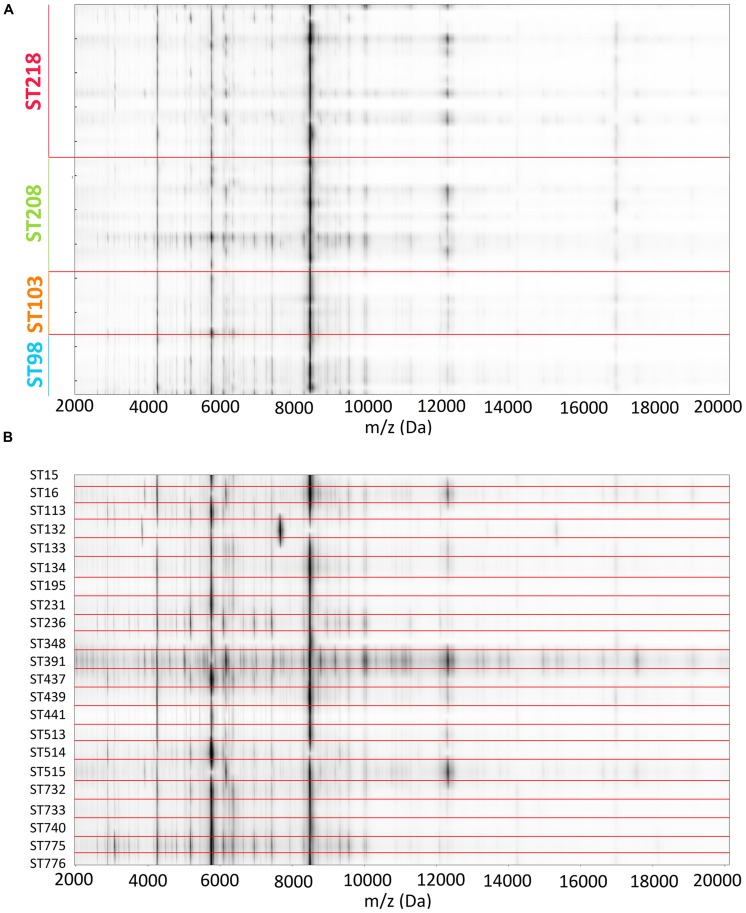
**Pseudo gel view representation obtained from the average mass spectra of each *Acinetobacter baumannii* isolate of ST98, ST103, ST208, and ST218 **(A)** and of *A. baumannii* isolates of the international collection (B)**.

Mean mass profiles of the isolates belonging to the four studied STs are presented in **Figure 1**. A very similar peak pattern can be found among the isolates of a single ST (data not shown) and among the four STs with almost no differences between them. The common peaks found among the four STs are summarized in the figure. **Figure 2** exhibits the pseudo gel views generated with MicrobeMS software considering all the isolates included in this study (ST98, S103, ST208, ST218 plus the 22 STs of the international collection). A high degree of consistency in the peak pattern can be found among the isolates of a single ST but also among the four STs (**Figure 2A**). It was impossible to obtain any degree of isolate discrimination according the ST solely based in the presence and/or absence of specific mass peak profiles even considering isolates epidemiologically unrelated and belonging to diverse STs (**Figure 2B**).

### Peakfind Function of Matlab

**FIGURE 3:**
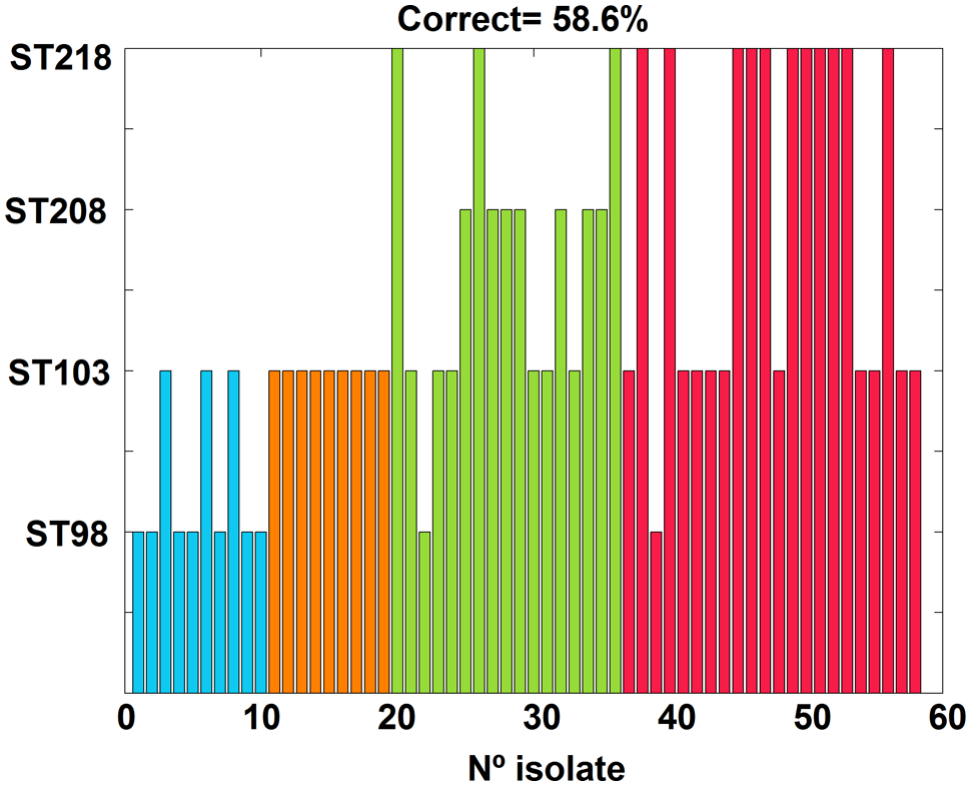
**Sequence type assignments for all isolates considering the spectral matching method.** Colors identify the real ST of each isolate and bars correspond to the method’s ST predictions (58.6% of correct predictions). Legend: 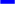 ST98, 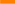 ST103, 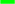 ST208, and 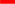 ST218.

Supplementary Figure S1 summarizes the peak positions founded with the *peakfind* function in the mass spectra from all isolates. No peaks were found above 17 kDa. Similarly to the observations in the pseudo gel view analysis, a very consistent peak pattern was observed among all the isolates with a low inter and intra-ST spectral variability. The comparison of each isolate peak profile with the four mean-ST peak profiles (see Materials and Methods) was used for estimating the ST of each isolate, **Figure 3**. Nevertheless, it was only possible to correctly predict the ST for 58.6% of the isolates. ST103 isolates were always correctly predicted; however, 19/58 isolates were erroneously predicted as ST103, meaning a high sensitivity (9/9 = 100%) and low specificity (30/51 = 58.8%) for the ST103 prediction. Moreover, it was possible to correctly predict 70% of the ST98 isolates; 41.2% of the ST208 isolates and 50% of the ST218 isolates.

### Partial Least Squares Discriminant Analysis

**FIGURE 4:**
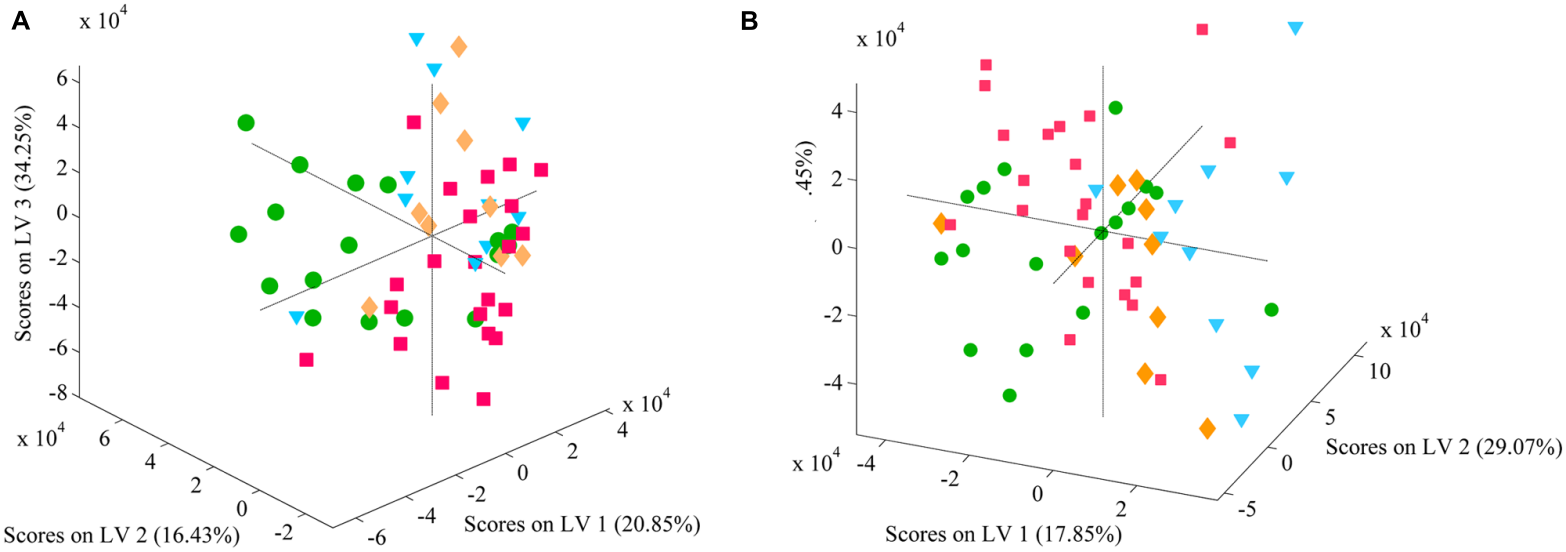
**Score plot corresponding to the first three latent variables (LVs) of the partial least squares discriminant analysis (PLSDA) regression model using the entire, 2–20 kDa, **(A)** and a selected, 4–10 kDa, **(B)** spectral range.** Legend: 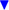 ST98, 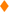 ST103, 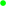 ST208, and 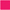 ST218.

The PLSDA models developed considering the entire (2–20 kDa) and a selected (4–10 kDa) spectral range are presented in **Figures 4A,B**, respectively. Considering the entire spectral range (**Figure 4A**) it was not possible to clearly discriminate the four STs as no individualized clusters could be found in the score map of the model. The PLSDA model was able to correctly predict the STs of 64.6% of the isolates (**Table 2**) being ST103 the one with a larger percentage of correct predictions (75.7%). The worst cases were observed for ST98 and ST208 for which plus than 40% of the isolates were erroneously predicted. Similarly, the PLSDA model obtained with the selected spectral range (4–10 kDa) did not allow the discrimination of the four STs (**Figure 4B**) still being ST103 the best predicted (73.7%), **Table 2**. Moreover, the total percentage of correct predictions was slightly higher (65.8%) for each of the three remaining STs considering the 4–10 kDa range.

**Table 2:** Confusion matrix for both of the *A. baumannii* PLSDA discrimination models, considering 20 LV (values are in %).

		ST obtained with MLST^(a)^							
		Spectral range 2–20 kDa				Spectral range 4–10 kDa			
		ST98	ST103	ST208	ST218	ST98	ST103	ST208	ST218
ST predicted by MALDI-TOF MS	ST98	56.2	5.7	12.6	14.4	56.6	8.1	13.1	12.7
	ST103	3.5	75.7	5.3	2.3	2.8	73.7	4.7	3.2
	ST208	17.2	8.8	56.8	13.7	19.5	10.0	60.6	13.6
	ST218	23.1	9.8	25.3	69.6	21.1	8.2	21.5	70.4

^a^Sequence type determined according with “Oxford” scheme (http://pubmlst.org/abaumannii/).

Partial least squares discriminant analysis models were also developed (whenever possible and according to the available information, please see **Table 1**) to correlate the mass profiles of the isolates with the results obtained from less discriminatory typing methods as AFLP, SG, and MLST scheme of Institute Pasteur. However, the clustering analysis was not congruent with any of these methods (data not shown).

## Discussion

In the last years an impressive growing number of studies unveiling the MALDI-TOF MS potential for routine bacterial species identification has been published (Böhme et al., 2010; Carbonnelle et al., 2010; Branquinho et al., 2014a,b; Sousa et al., 2014a). However, the suitability of this mass spectrometry technique for bacterial discrimination at the subspecies level has been barely explored, with contradictory outcomes for particular species (Barbuddhe et al., 2008; Dieckmann and Malorny, 2011; Wolters et al., 2011; Gekenidis et al., 2014; Lasch et al., 2014; Novais et al., 2014). The goal of this work was to assess the ability of MALDI-TOF MS to depict *A. baumannii* clones, with particular interest for the worldwide spread lineages (ST98, ST103, ST208, and ST218), contributing to a better understanding of the capabilities and limitations of this technique in bacterial typing. Analysis of the mass spectra of the four STs was attempted using three approaches and revealed some mass-to-charge ratios already identified as *Acinetobacter* genus and *A. baumannii* species-specific (Böhme et al., 2010; Espinal et al., 2011; Šedo et al., 2013; Sousa et al., 2014a). However, the high similarity among mass profiles of the *A. baumannii* lineages analyzed prevented the STs discrimination either by peak pattern direct analysis (**Figure 1**) or based on the presence/absence of specific peaks depicted in the pseudo gel views (**Figure 2A**). Moreover, attempting to assign a ST based on the comparison of each isolate’s mass profile with the mean-ST profile also resulted in a low percentage of correct identifications (58%, **Figure 3B**). Previous studies, including from our group (Novais et al., 2014; Sousa et al., 2014a), have demonstrate that the use of specific and optimized chemometric tools in MALDI-TOF MS data analysis improves the bacterial discrimination derived from this spectroscopic methodology. In this context, we attempted to discriminate the isolates with a PLSDA analysis considering two distinct mass ranges (**Figures 4A,B**). Although the degree of discrimination slightly improved, only 64.6 and 65.8% of correct STs predictions were obtained for the two considered mass ranges, demonstrating the current inadequacy of MALDI-TOF MS for discrimination of major *A. baumannii* STs. The difficulty to differentiate these STs based on MALDI-TOF MS analysis could possibly be associated with the relatedness of their allelic profiles. In fact, ST98 is a double locus variant of ST208 and ST218 in *gyrB* and *gpi* and ST208 a single locus variant of ST218 in the *gpi* allele. Despite the low ability to discriminate the four STs, *A. baumannii* ST103 isolates always presented the higher rate of correct ST predictions whether considering the comparison of the ST-mean mass profiles or the chemometric approach. It is of note that the allelic profile of ST103 isolates is the most dissimilar one, presenting only one common allele with ST98, the *gpi* one^3^. This fact could contribute to a more dissimilar ribosomal protein/peptide profile of ST103 isolates and its subsequent higher rate of correct identifications. The difficulty to differentiate these four STs based on their mass spectra suggests that these clones possess a very similar profile in what concerns to the molecules routinely observed in these MALDI experiments.

As a high throughput technique, MALDI-TOF MS competes with other spectroscopic techniques as Raman (Maquelin et al., 2006), and Fourier Transform Infrared Spectroscopy (FTIR) for bacterial typing at different taxonomic levels. It is of note, the suitability of FTIR to discriminate *A. baumannii* lineages, including the STs included in this study (Sousa et al., 2014b), which is also a sensitive, quick and low cost technique. Nevertheless, with the recognition of the interest in the microbiological diagnostic of MALDI-TOF MS, associated with the increasing availability of MALDI-TOF MS equipment in routine laboratories, there is a particular interest on methodology developments assisting bacterial epidemiology. In this way, further assays testing the ability of MALDI-TOF MS to discriminate *A. baumannii* lineages with different sample preparation conditions and matrix solutions should be conducted. It also should be noted that, despite our MALDI data had been compared with four distinct classification methods (two different MLST schemes, AFLP and SG), presenting different typing resolutions, it was not congruent with the grouping obtained with any of these approaches. In this way, it does not offer, in these experimental conditions, an advantage over other rapid methods such as DiversiLab rep-PCR-based typing, trilocus sequence-based typing, or single-locus-sequence-based typing of *bla*_OXA-51_-like genes. However, we do not exclude the possibility that other classification method could somehow fit MALDI-TOF MS data.

## Conclusion

In this work we evaluate the ability of MALDI-TOF MS to discriminate *A. baumannii* clones. This mass spectroscopic technique, which revealed in previous studies a high discrimination power for species identification within the *A. calcoaceticus–A. baumannii* complex, demonstrates an insufficient result when used for discrimination at the clonal level. These findings suggest that the detected molecules, mainly ribosomal peptides and/or proteins, remain unchanged during clonal diversification in this species. Further studies, namely using different sample preparation conditions, are needed to provide further insights on the suitability of MALDI-TOF MS for typing *A. baumannii* at a subspecies level.

## Conflict of Interest Statement

The authors declare that the research was conducted in the absence of any commercial or financial relationships that could be construed as a potential conflict of interest.

## Footnotes

1 http://pubmlst.org/abaumannii/

2 http://www.mara-ms.com

3 http://pubmlst.org/abaumannii/
