# Supplementary figures and images for: Unsuitability of MALDI-TOF MS to discriminate Acinetobacter baumannii clones under routine experimental conditions
Source: Front Microbiol. 2015 May 19;6:481. doi: 10.3389/fmicb.2015.00481 (PMC4436932; doi:10.3389/fmicb.2015.00481)

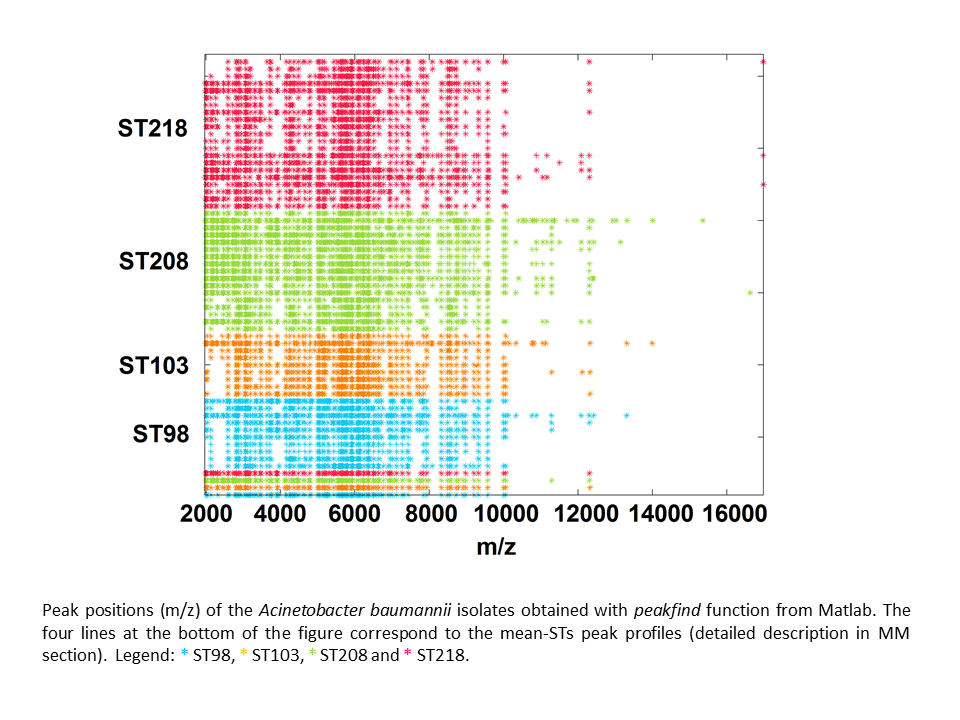

Supplement: Supplementary file 1 [file Image_1.TIF]
